# Development of an enzyme-linked immunosorbent assay (ELISA) for determining neutrophil elastase (NE) – a potential useful marker of multi-organ damage observed in COVID-19 and post-Covid-19 (PCS)

**DOI:** 10.3389/fmolb.2025.1542898

**Published:** 2025-02-25

**Authors:** Joanna Adamiec-Mroczek, Joanna Kluz, Sandra Chwałek, Maciej Rabczyński, Kinga Gostomska-Pampuch, Łukasz Lewandowski, Marta Misiuk-Hojło, Beata Ponikowska, Goutam Chourasia, Ilias Dumas, Andrzej Gamian, Żanna Fiodorenko-Dumas, Bogusława Konopska, Agnieszka Gola, Klaudia Konikowska, Daniel Strub, Agnieszka Bronowicka-Szydełko, Katarzyna Madziarska

**Affiliations:** ^1^ Clinical Department of Ophthalmology, Wroclaw Medical University, Wroclaw, Poland; ^2^ Clinical Department of Diabetology, Hypertension and Internal Disease, Wroclaw Medical University, Wroclaw, Poland; ^3^ Faculty of Chemistry, Wroclaw University of Science and Technology, Wroclaw, Poland; ^4^ Department of Biochemistry and Immunochemistry, Wroclaw Medical University, Wroclaw, Poland; ^5^ Department of Physiology and Pathophysiology, Wroclaw Medical University, Wroclaw, Poland; ^6^ Department and Clinic of Emergency Medicine, Wroclaw Medical University, Wroclaw, Poland; ^7^ Department of Clinical Physiotherapy and Rehabilitation, Wroclaw Medical University, Wroclaw, Poland; ^8^ Laboratory of Medical Microbiology, Hirszfeld Institute of Immunology and Experimental Therapy, Polish Academy of Sciences, Wroclaw, Poland; ^9^ Diagnostic Scientific and Teaching Laboratory, Department of Laboratory Diagnostics, Wroclaw Medical University, Wroclaw, Poland; ^10^ Department of Physical Chemistry and Biophysics, Wroclaw Medical University, Wroclaw, Poland; ^11^ Department of Dietetics and Bromatology, Wroclaw Medical University, Wroclaw, Poland

**Keywords:** COVID-19, ELISA, neutrophil elastase 1, post-COVID-19-syndrome (PCS), long-COVID biomarkers

## Abstract

**Background:**

The ongoing post-COVID-19 syndrome (PCS) epidemic, causing complications of diverse etiology, necessitates the search for new diagnostic markers and the development of widely accessible methods for their detection. This would enable the prognosis of PCS progression and faster implementation of targeted treatments. One potential marker is neutrophil elastase (NE), whose elevated levels in the blood during PCS may result from organ damage caused by increased secretion of severe inflammatory mediators or amyloidosis resulting from the interaction of NE with SARS-CoV-2. The aim of this publication is to present a step-by-step method for designing an enzymatic ELISA test, enabling the quantitative assessment of NE in the blood serum of patients.

**Methods:**

NE was measured using the designed ELISA test.

**Results:**

The study outlines all the steps necessary for designing and optimizing the ELISA test, including the selection of standards, primary and secondary antibodies, and their dilutions. Using the test, elevated NE levels were demonstrated in patients with advanced-stage diabetic nephropathy after symptomatic COVID-19, compared to a relative group of patients sampled before COVID-19.

**Conclusion:**

The undertaken efforts enabled the development of a test with high performance parameters (initially set sensitivity: ≥40 pg/μL; intra-assay precision: 7%; inter-assay precision <20%). No significant cross-reactivity with other tested proteins was observed. Serial dilution of plasma samples resulted in a proportional decrease in signal intensity.

## 1 Introduction

The Coronavirus Disease 2019 (COVID-19) pandemic has highlighted the reality of the emergence of multireceptor viruses, such as Severe Acute Respiratory Syndrome Coronavirus 2 (SARS-CoV-2), which, through interactions with various cell types, can disrupt their metabolism and, in more severe cases, lead to their degradation. A strong interaction between the virus and the cell is particularly likely when the cell expresses genes encoding proteins that facilitate viral fusion with the cell (main protease TMPRSS2) (Qu et al., 2022) and attachment and entry into the cell for replication purposes (the Angiotensin-Converting Enzyme 2 receptor, ACE2) (Li et al., 2020). Since ACE2 receptors are present on cells of various organs, such as the thyroid, pancreas, testes, lung epithelium, blood vessels of the kidneys, and cardiac muscle, COVID-19 may manifest not only with pulmonary symptoms but also extrapulmonary ones (Li et al., 2020). Consequently, the replication of SARS-CoV-2 can lead to the overexpression of genes encoding proteins involved in various cellular processes, including Janus kinase (JAK), signal transducer and activator of transcription (STAT), nuclear factor kappa-B (NF-κB), and mitogen-activated protein kinase (MAPK), which induce cytokine secretion (Clausen et al., 2021). Excessive cytokine activation can trigger the so-called “cytokine storm” (Rossini et al., 2023), often leading to cell apoptosis, fostering the development of systemic inflammation, and causing organ damage (Clausen et al., 2021). Cell lysis results in the release of proteins into the bloodstream that are either not normally present or are typically observed at low concentrations (or activity levels, in the case of enzymes). The type of released proteins depends on the type of cell undergoing lysis, which is why these proteins are considered selective markers of specific metabolic disturbances (Rabczyński et al., 2024).

An example of an intracellular protein released during severe trauma or acute infection, such as SARS-CoV-2, is neutrophil elastase (NE). NE belongs to the family of serine proteases. Physiologically, it is present in neutrophils and extracellular neutrophil traps, sites not only for pathogen neutralization but also for the accumulation of erythrocytes, platelets, and fibrin. These components form clots during acute inflammation, subsequently inducing the extrinsic coagulation cascade (Nie et al., 2020). In the lungs, NE activity degrades collagen and elastin fibers, damaging the extracellular matrix, which leads to excessive mucus secretion and impaired ciliary movement (Silva and Radic, 2022). Additionally, NE is involved in the cytokine storm via two pathways: it induces cytokine secretion (e.g., IL-8, IL-1ß) (Scheller et al., 2021) and is itself induced by cytokine activity. The presence of NE in the blood indicates neutrophil or neutrophil trap damage due to trauma, inflammation, or infection. High NE activity in the blood can result in multi-organ damage, thrombosis, and exacerbation of COVID-19 (Hazeldine and Lord, 2021). These observations make NE a potentially significant component in designing a predictive model for post-COVID syndrome (PCS), a condition of diverse etiology declared by the World Health Organization (WHO) as the next epidemic of the 21st century (Raman et al., 2022).

So far, few studies have been conducted that indicate a relationship between NE and PCS, primarily due to the fact that the COVID-19 pandemic ended relatively recently, and intensive work is currently underway to identify potential markers of PCS. These markers are mainly sought among proteins whose elevated concentrations were observed during the acute phase of COVID-19, such as neuron-specific enolase or neutrophil elastase. The release of extracellular neutrophil traps (NETs) carrying NE and myeloperoxidase in the lower respiratory tracts of patients with severe COVID-19 is associated with acute damage to the alveoli (Ackermann et al., 2021; Ouwendijk et al., 2021; Wang et al., 2023). Furthermore, the path mechanism of elastin lysis catalyzed by NE in activated neutrophils leads to the release of strong mediators of inflammation and interstitial fibrosis (Starcher and Peterson, 1999) and is linked to injury-associated fibrosis and chronic obstructive pulmonary disease (COPD) (de Brouwer et al., 2018; Woodruff et al., 2023; Narasaraju et al., 2024) showed that plasma samples from hospitalized COVID-19 patients had approximately 30-fold higher NE-A1AT (alpha-1-antitrypsin) complexes than plasma from healthy donors, suggesting that NE or such complexes may provide an indication of host tissue damage due to inflammation in respiratory lung infections. It has also been observed that NE levels in patients with advanced diabetic nephropathy related to T2DM, who had symptomatic COVID-19, were significantly higher than in serum samples from patients with advanced diabetic nephropathy related to T2DM, collected before the COVID-19 pandemic (Rabczyński et al., 2024), immune-mediated fibrosis and ongoing neutrophil activity are associated with an inflammatory state that persists for more than a year after the acute phase of SARS-CoV-2 infection. Furthermore, in *in vitro* studies, it has been shown that the reaction between the Spike protein and NE results in the formation of amyloid-like fibrils. NE efficiently cleaved S-protein, rendering exposure of amyloidogenic segments and accumulation of the most amyloidogenic peptide of spike protein. This is a potential mechanism which may lead to amyloidosis in human and consequently to long-COVID (Nyström and Hammarström, 2022).

The aim of this publication is to present a step-by-step guide to developing an enzyme-linked immunosorbent assay for quantitatively assessing a specific protein in patient blood sera. Using self-developed ELISA assays not only significantly reduces the cost of testing (compared to ready-to-use kits) but, more importantly, allows control over each stage of the process. Consequently, this enables verification that the visualized results truly reflect the antigen–primary antibody reaction. The protein measured in the developed test was neutrophil elastase (NE), which was quantitatively determined in the blood sera of patients with T2DM who had symptomatic COVID-19, with and without advanced diabetic nephropathy. This allowed us to assess whether the test would enable the identification of statistically significant differences in NE levels between patients with T2DM without advanced nephropathy and those with advanced diabetic nephropathy, as demonstrated in (Rabczyński et al., 2024), where the potential diagnostic significance of NE in PCS diagnostics was highlighted. The aim of the study is also to validate the developed ELISA test by determining the values of parameters characterizing enzyme-linked immunosorbent assay, such as the detection range, test sensitivity, inter-assay CV, intra-assay CV, signal-to-noise ratio (S/N), limits of detection (LoD), the lower limit of quantification (LLOQ), the upper limit of quantification (ULOQ), and to compare the test with other commercial assays.

## 2 Materials and methods

The following equipment were used in the developed ELISA test: Multichannel and single-channel manual pipettes (F1-ClipTip™ Manual Pipettes, Thermo Scientific™, Massachusetts, GA, United States), plates (Nunc MaxiSorp®, Thermo Fisher Scientific, Massachusetts, GA, United States), absorbance reader: BioTek Synergy H1 Multimode Reader (Agilent, Santa Clara, CA, United States), incubator (Memmert INP 500 Incubator, LabMakelaar Benelux B.V., Netherlands), centrifuge (Benchmark Scientific, BENCHMIXER™ VORTEXER MIXER, Sayreville, NJ 08872 US), microplate washer (Biosan–InteliWasher 3D-IW8, Microplate Washer, Stargard, Poland).

In the designed immunoassay, primary and secondary antibodies play a key role. Due to their high specificity and affinity, the following primary and secondary antibodies were selected: Mouse Anti-Human Neutrophil Elastase Monoclonal IgG1 Antibody (ELA10, 101.5, No: # MA1-10608, ThermoFisher Scientific, Massachusetts, GA, USA), Goat Anti-Mouse IgG1 (HRP PA1-74421, ThermoFisher Scientific, Massachusetts, GA, USA). Chosen primary antibodies detects neutrophil elastase from human samples and has been successfully used in ELISA and in studies of inhibition and enzyme kinetics of neutrophil elastase (Floyd et al., 2016). When selecting secondary anti-mouse IgG antibodies, the primary focus was to ensure that they do not cross-react with human IgG (false-positive results) and that they are specifically directed against mouse IgG1. The selected secondary antibodies had already been used by other research teams for detection, sorting, or purification (Cui et al., 2024; Fernandez et al., 2024; Sobczak et al., 2024).

To obtain the most accurate and reproducible test results, optimization of the blocking stage is essential. Since the choice of the appropriate blocking agent depends on various factors, including the type of sample, detection system used, and the proteins present in the tested material, it is necessary to verify whether any interactions occur between the blocking agent and components of the test. Therefore, the test evaluated the effectiveness of three commonly used blocking agents: skimmed milk, casein, and albumin [(skimmed milk powder (250g SM, Poland), casein from bovine milk (C7078, Merck, Darmstadt, Germany), bovine serum albumin diagnostic grade, powder (820451, Merck, Darmstadt, Germany)]. The most suitable blocking agent was found to be a 7.5% skimmed milk solution. An added value of this blocking agent was its concentration, which was below 10%. Skimmed milk is successfully used in ELISA tests (Bronowicka-Szydełko et al., 2021). In addition, the following reagents were used: human neutrophil elastase (CAS 9004-06-2, No. 324681-50UG, Merck, Darmstadt, Germany), Tris-Buffered Saline, 0.1% Tween 20 Detergent (Merck, Darmstadt, Germany, TBS-T), o-phenylenediamine dihydrochloride (OPD; Merck, Darmstadt, Germany).

The same biological material described in (Rabczyński et al., 2024) was used for designing and optimizing the ELISA test. This material consisted of blood sera obtained from patients with type 2 diabetes who had experienced COVID-19 and were treated at the Clinic of Angiology, Diabetology, and Hypertension of the Jan Mikulicz-Radecki University Clinical Hospital in Wrocław, as well as from patients with stage V diabetic nephropathy, who were treated at the Clinic of Nephrology, Transplantology, and Internal Diseases. The study included only patients who had experienced symptomatic COVID-19. Blood was collected from individuals aged 45–89 years, maintaining a female-to-male ratio of 1:1. The material obtained from patients was processed as quickly as possible, within 1 hour of collection, properly secured, anonymized, and stored at −80°C. Approval for the collection of biological material was granted by the Bioethics Committee of Wrocław Medical University (No. KB 187/2019; KB 780/2022, KB).

### 2.1 Scheme of development and optimization of our own ELISA assay for quantitative assessment of NE in blood serum

To develop an ELISA immunoenzymatic assay for the quantitative assessment of NE levels in patient blood sera, the first step was to select the components of the designed test, specifically: Primary antibodies specific to human NE, secondary antibodies that recognize the primary antibodies (taking into account the species from which the primary antibodies were obtained) and do not cross-react with human immunoglobulins or other serum proteins, and human native NE derived from neutrophils, allowing for the preparation of a standard curve.

**FIGURE 1:**
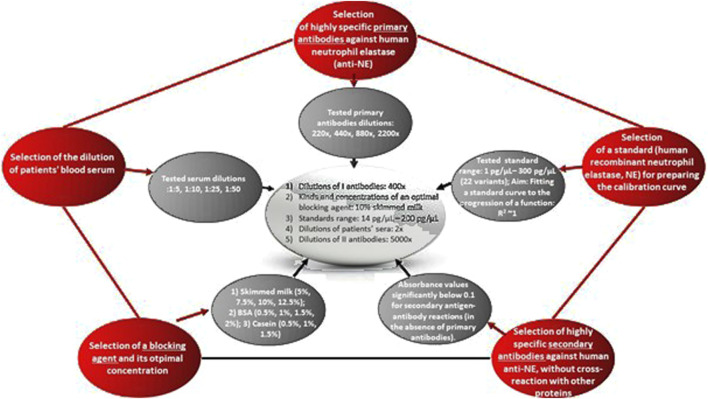
Scheme of the designed ELISA assay for the quantitative assessment of neutrophil elastase in blood serum.

The designed ELISA test was conducted according to the procedure outlined in Figure 1. First, a sample of blood serum or a properly prepared standard (100 µL/well in triplicate), suspended in phosphate-buffered saline (PBS, pH 7.4), was added to an ELISA plate and incubated at 37°C for 4 h. Next, the solutions were removed, the plate was washed three times with PBS-T buffer (300 µL/well), and blocked with a 5% skimmed milk solution. The plate was incubated with the milk solution overnight at 4°C. Following this, the milk solution was removed, the plate was washed as before, and 100 µL/well of primary antibody solution specific for human neutrophil elastase (NE) was added. The plate was incubated with the primary antibodies for 2.5 h at 37°C, after which the excess solution was removed, and the plate was washed as previously described. In the next step, the wells were treated with 100 µL/well of secondary antibody solution specific for NE. The plate was incubated with the secondary antibodies for 1.5 h at 37°C, followed by the removal of the excess solution and washing of the plate as described above. The wells were then treated with a substrate solution containing o-phenylenediamine dihydrochloride (OPD) for horseradish peroxidase (HRP), to enable a colorimetric reaction, in a volume of 100 µL/well. The plate was incubated with OPD for 20 min at room temperature in the dark, and absorbance values were subsequently read at λ = 450 nm.

In the designed test, each step and each of the three key analytes were meticulously controlled. For example, secondary antibodies conjugated with the enzyme (horseradish peroxidase, HRP) were tested to ensure they did not produce false-positive signals due to accidental cross-reactivity with human immunoglobulins (IgG) or other proteins potentially present in patient blood sera. This verification was performed despite selecting secondary antibodies specifically designed not to cross-react with human IgG. Similarly, to determine the appropriate concentrations of standards required for the calibration curve, 22 variations of concentrations of human native NE, obtained from human neutrophils and suspended in carbonate buffer (pH 9.6), were tested. Additionally, various dilutions of blood sera in PBS (pH 7.4) and dilutions of primary antibodies were evaluated. Half of the plate was designated, for example, as a control for the secondary antibodies, while the other half was used for optimizing the standard concentration.

## 3 Results

Developing an own method for measuring the concentration of a specific protein would enable control over each step of the ELISA test and its optimization. It is crucial to ensure that the visualized enzymatic reaction result is due to the interaction of the secondary antibody with the primary antibody, rather than the secondary antibody reacting with a serum protein. Therefore, the initial step involved verifying that the secondary antibodies exclusively reacted with the primary antibodies and not with other serum components, despite the secondary antibody manufacturer assuring no reactivity with human immunoglobulins. To validate this stage of the ELISA test, plates coated with four different dilutions of sera from four patients were treated with both primary and subsequently secondary antibody solutions. Parallel controls were conducted where these sera were treated solely with the secondary antibody solution. The reactions were then triggered, and absorbance values were measured.

**FIGURE 2:**
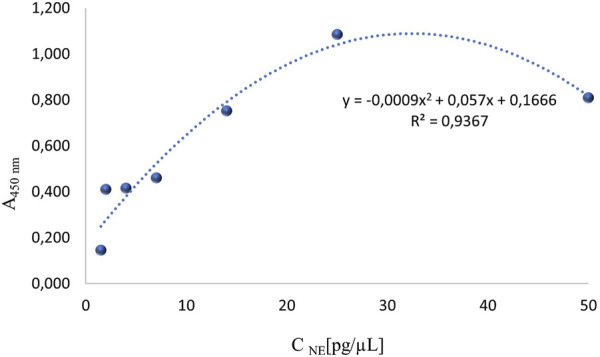
The first standard curve for the quantitative assessment of neutrophil elastase, which required optimization.

**FIGURE 3:**
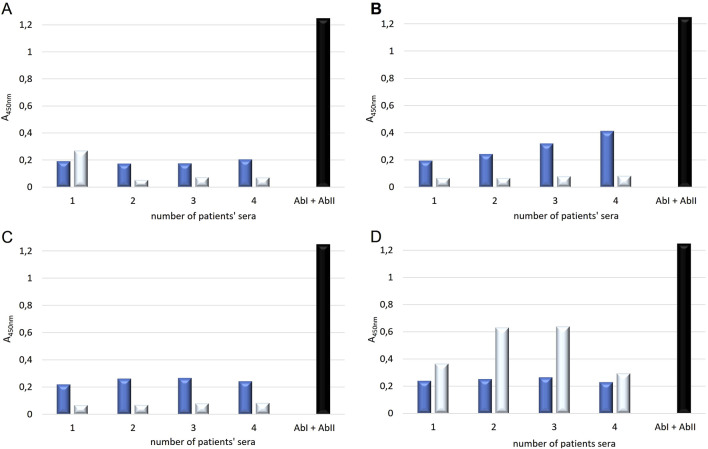
The absorbance values obtained in the custom-designed ELISA test for NE determination for four patient sera in dilutions: **(A)** 5x, **(B)** 10x, **(C)** 25x, **(D)** 50x, treated with IAb and IIAb (dark blue color) and IIAb (light blue color) – serving as the control for IIAb, BLANK (black color).

On a 96-well plate, standards and sera diluted 5-fold, 10-fold, 25-fold, and 50-fold were applied. Simultaneously, reactions without primary antibodies were conducted to check whether the secondary antibodies exhibited nonspecific reactivity with proteins in the patients’ sera. The standard curve is presented in Figure 2. The generated curve had a parabolic shape, and the R^2^ value of approximately 1 indicated a good fit of the curve to the obtained values. Based on the resulting curve, it was possible to select appropriate dilutions of the NE standard so that the obtained curve included the potential reference range. The absorbance values obtained for four different dilutions of serum from four patients and their controls are shown in Figure 3.

Based on the obtained values for the tested sera (treated with I and II antibodies) and control sera (the same sera treated only with II antibodies), significantly lower absorbance values were observed for the control samples compared to the tested groups for sera diluted 5x, 10x, and 25x. The 50-fold dilution proved to be too high, and the opposite phenomenon, resulting from nonspecific interactions, was observed. An abnormal relationship was observed for the reaction background (blank) – the absorbance values were significantly higher than for the samples containing serum. It was concluded that it is necessary to attempt to determine the NE concentration at which the obtained absorbance values result solely from the NE reaction with the primary antibodies, without interference from cross-reactions with secondary antibodies or binding of the secondary antibodies to the well surface.

**FIGURE 4:**
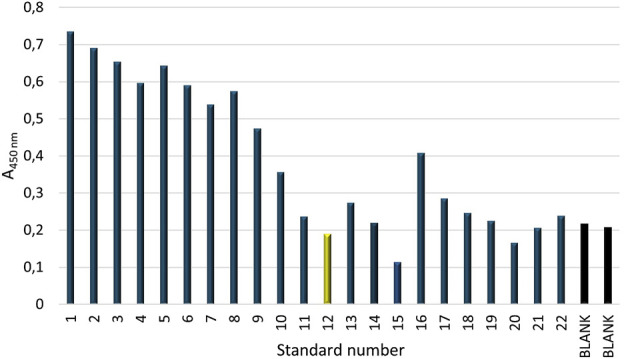
Absorbance values for the individual concentrations of the standard (human native neutrophil elastase) – samples 1-22, and for the background (BLANK); standard number: 1-22 - in the order of increasing standard dilutions. The main steps of the ELISA test were carried out under the following conditions: I) Incubation with AbI: 440x, 2.5 h, 37°C; II) blocking solutions: 5% skimmed milk, overnight, 4°C; III) AbII: 5000x, 1.5 h, 37°C.

For this purpose, an ELISA test was designed to assess the reactivity of a series of dilutions of the standard solution of human native neutrophil elastase, obtained from human neutrophils (1–300 pg/mL), with both primary and secondary antibodies, as well as to control the series of dilutions of the standard solution of human native neutrophil elastase (1–300 pg/mL) with secondary antibodies. The test also attempted to determine the absorbance values for the background (BLANK), i.e., the samples without the standard. A 96-well plate was used to apply 22 standard solutions with different concentrations in the range of 300 pg/μL – 1 pg/μL. In addition to the standards, the plate also contained a blank sample. Each standard solution and blank was applied in two replicates. The absorbance values for each concentration of the standard (human native neutrophil elastase), i.e., for samples 1–22, and for the background (BLANK) are shown in Figure 4.

**FIGURE 5:**
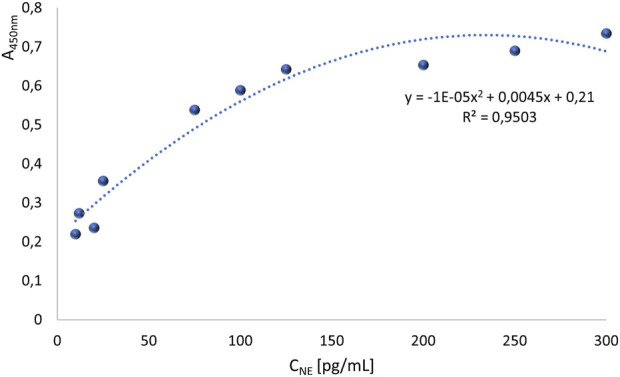
Standard curve for quantitative assessment of neutrophil elastase in blood serum (for concentration range of standard: 20 pg/mL to 300 pg/mL) under the given ELISA test conditions of the main steps: I) Incubation with AbI: 440x, 2.5 h, 37°C; II) blocking solutions: 5% skimmed milk, overnight, 4°C; III) AbII: 5000x, 1.5 h, 37°C.

The obtained absorbance values revealed disturbances that appeared at low concentrations of NE–some values were surprisingly high at low standard concentrations. For example, at a concentration of 7 pg/μL (sample 16), the absorbance was 0.468, and for a concentration of 8 pg/μL (sample 15), the absorbance was 0.169. Based on the obtained results, it was concluded that the detection limit (for NE concentration) under the test conditions was the concentration of standard 12, which is 15 pg/μL. Absorbance values for solutions with lower standard concentrations were found to be erroneous, likely due to cross-reactivity, possibly resulting from insufficient blocking of the ELISA plate wells. Therefore, the absorbance values for samples 1-11 were considered reliable, and a standard curve was constructed for these samples, as shown in Figure 5. The standard curve prepared for standards corrected by corresponding control values for concentrations of 20 pg/mL to 300 pg/mL, shown in Figure 5, exhibited a relatively high determination value (R^2^ = 0.95), indicating that the curve was well fitted to the progression of the function.

**FIGURE 6:**
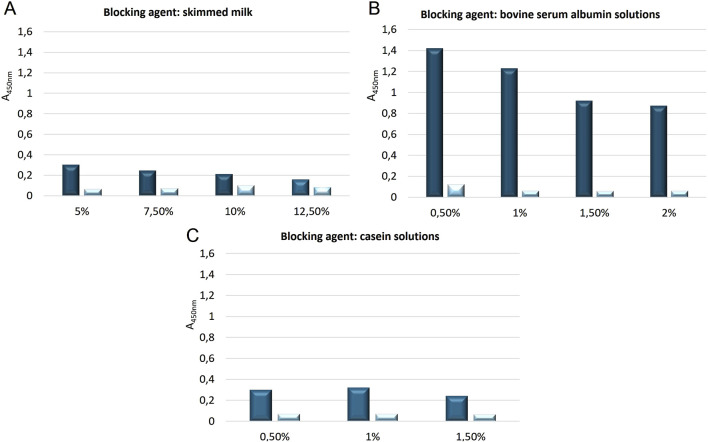
The absorbance values of the ELISA tests conducted with three different blocking agents at various concentrations (marked in light blue), alongside the control reaction (i.e., the plate without primary antibody solution–secondary antibody control), are as follows: **(A)** Powdered skimmed milk at concentrations of 5%, 7.5%, 10%, and 12.5%; **(B)** Bovine serum albumin solutions at concentrations of 0.5%, 1%, 1.5%, and 2%; **(C)** Casein solutions at concentrations of 0.5%, 1%, and 1.5%.

Due to the still relatively high background absorbance (BLANK) values, an experiment was conducted to test the effectiveness of blocking the plate with various solutions commonly used in ELISA tests: powdered skimmed milk (at concentrations of 5%, 7.5%, 10%, and 12.5%), albumin (at concentrations of 0.5%, 1%, 1.5%, and 2%), and casein (at concentrations of 0.5%, 1%, and 1.5%). For each factor, a control reaction was conducted on the same plate, where the blocking agent was applied with the same solutions except for the primary antibody solution. This allowed testing whether the secondary antibodies recognize proteins present in the blocking solutions. The results from these experiments, aimed at selecting the optimal blocking agent and its optimal concentration, are presented in Figure 6. Based on the conducted experiments, it was observed that low absorbance concentrations (indicating proper blocking of the plate) were only seen for the milk solution at ≥ 7.5% powdered skimmed milk. The other blocking agents showed significantly higher absorbance values, i.e., for casein solutions >0.25, and for albumin solutions >0.9. The absorbance values for all control reactions were low, i.e., <0.1.

**FIGURE 7:**
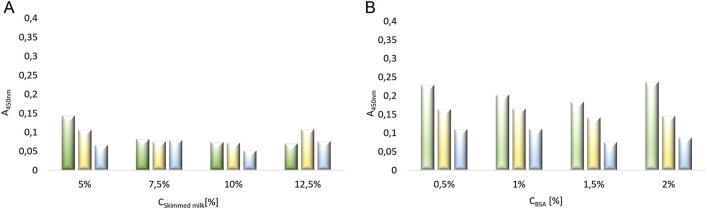
The absorbance values obtained in the ELISA test conducted with two different blocking agents at various concentrations: **(A)** powdered skimmed milk at concentrations of 5%, 7.5%, 10%, 12.5%; **(B)** bovine serum albumin solutions at concentrations of 0.5%, 1%, 1.5%, 2%, using three different dilutions of the primary antibodies (RAbI): 440x (green), 880x (yellow), 2200x (blue).

In the next stage, an attempt was made to determine the dilution of the primary antibody solution using two blocking agents: powdered skimmed milk and albumin solution. This experiment aimed to confirm the observations from the previous experiment regarding the selection of the blocking agent and to optimize the conditions for the stage of applying the primary antibodies to the plate. The following concentrations were used: powdered skimmed milk – 5%, 7.5%, 10%, 12.5%, and albumin – 0.5%, 1%, 1.5%, 2%, as well as primary antibody dilutions – 440x, 880x, 2200x. The absorbance results from the conducted experiment are presented in Figure 7. Based on the experiment, it was determined that the bovine serum albumin solution in the concentration range of 0.5%–2% for both 440x and 880x dilutions of the primary antibodies did not allow for obtaining an average absorbance value (measured at λ = 450 nm) below 0.15. This suggests the occurrence of nonspecific reactions between the primary antibodies and the albumin. The use of a 2200x dilution of the primary antibodies, although resulting in average absorbance values below 0.12, was most likely due to an excessive dilution of these antibodies, as indicated by the low absorbance values obtained for each of the bovine serum albumin concentrations used. The results obtained from using four different concentrations of skimmed milk (5%, 7.5%, 10%, 12.5%) as a blocking agent for three different dilutions of primary antibodies (440x, 880x, 2200x) showed that using a milk concentration of ≥7.5% allowed for obtaining average absorbance values <0.1 for each dilution of the primary antibodies (440x, 880x, 2200x). These results confirmed the observation from the previous experiment, indicating the effectiveness of skimmed milk blocking in the concentration range of 7.5%–12.5% in the designed ELISA test.

**FIGURE 8:**
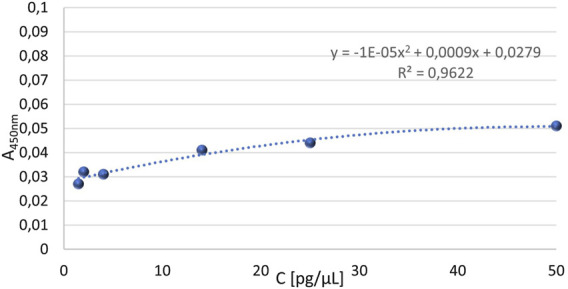
Standard curve for quantitative assessment of neutrophil elastase, corrected with control values, in the concentration range of standard: 1.5 pg/μL to 50 pg/μL. The main steps of the ELISA test were carried out under the following conditions: I) Incubation with AbI: 440x, 2.5 h, 37°C; II) blocking solutions: 7.5% skimmed milk, overnight, 4°C; III) AbII: 5000x, 1.5 h, 37°C.

**FIGURE 9:**
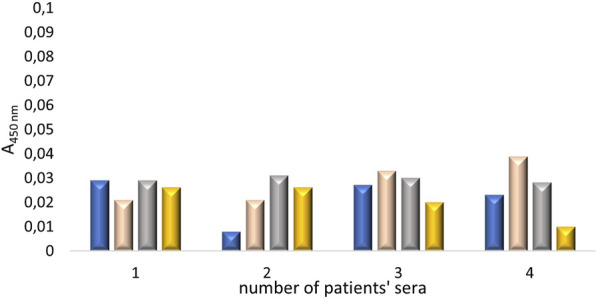
The absorbance values (corrected for the corresponding control absorbance values) obtained for the serum samples from four patients with advanced diabetic nephropathy at four different dilutions: 5x (blue), 10x (pink), 25x (gray), and 50x (yellow).

**FIGURE 10:**
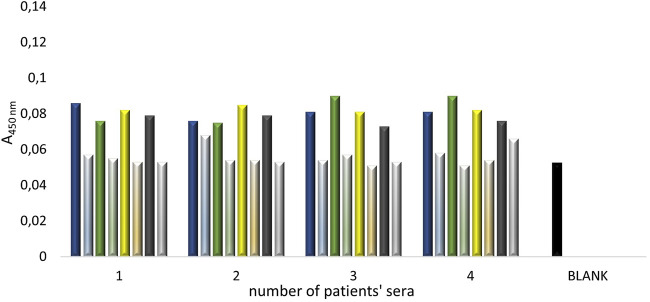
The absorbance values obtained for: the blood sera of four patients with advanced diabetic nephropathy at four different dilutions: 5x, 10x, 25x, and 50x, their corresponding controls, and BLANK (dark blue: 5x test sample; light blue: 5x control; dark green: 10x test sample; light green: 10x control; dark yellow: 25x test sample; light yellow: 25x control; black: 50x test sample; gray: 50x control; black: test control).

In the next stage, an attempt was made to optimize the dilutions of blood sera subjected to the ELISA test. The experiment was carried out using the conditions established in previous tests, i.e., blocking the plate with 7.5% skimmed milk and applying the primary antibodies diluted 440 times. Four different blood sera from patients with advanced diabetic nephropathy who had recovered from COVID-19 were tested, each at four different dilutions (5x, 10x, 25x, 50x). A control was performed for each dilution of the blood serum, using the same reagents, except for the primary antibodies. The plate was also coated with standard solutions of human native neutrophil elastase at the following concentrations: S1 = 50 pg/μL, S2 = 25 pg/μL, S3 = 14 pg/μL, S4 = 7 pg/μL, S5 = 4 pg/μL, S6 = 2 pg/μL, S7 = 1.5 pg/μL, as well as a blank (BLANK) – containing neither blood serum nor NE standard. Based on the results, a standard curve for NE was prepared, corrected with the control values of the standards. The generated standard curve (Figure 8) showed a relatively high determination value of R^2^ (0.962), indicating that the curve was well-fitted to the function. However, it was observed that the curve progressed within the range of corrected absorbance values from 0.021 to 0.050, which are relatively low, suggesting that the dilution of the primary antibodies might be too high. The absorbance values for the given dilutions of the patient serum, corrected for the corresponding absorbance values of the control samples, were then visualized, as shown in Figure 9. Based on the obtained absorbance values for the blood sera of four patients with advanced diabetic nephropathy at four different dilutions: 5x, 10x, 25x, and 50x, corrected for the corresponding control absorbance values, it was found that the 5-fold, 10-fold, and 25-fold dilutions of the blood sera allowed for the detection of NE concentrations in all serum samples. The experiment also showed that the absorbance value for the BLANK was lower than the absorbance values for both the test samples and their corresponding controls (Figure 10), indicating that the conditions for the ELISA test were properly optimized.

**FIGURE 11:**
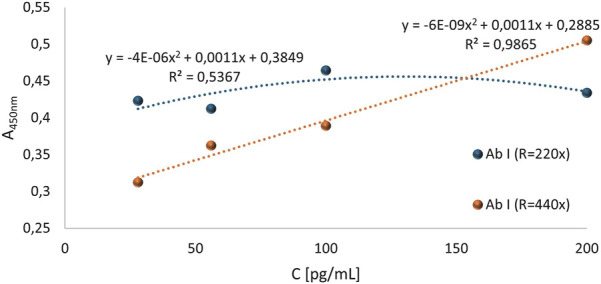
Standard curves for human native neutrophil elastase determined based on absorbance values obtained using primary antibodies diluted 220-fold and 440-fold.

Based on the conducted experiment, it was concluded that the standard curve allowed for the determination of NE concentrations in blood sera diluted 5-fold, 10-fold, or 25-fold. Furthermore, with these selected test conditions, the desired absorbance values for BLANK were obtained, i.e., lower than the absorbance values for the blood serum samples and their corresponding controls. However, relatively low absorbance values were obtained for all serum dilutions. Therefore, it was decided to reduce the dilution range of the primary antibodies and apply a higher concentration range for the standards in the next test. Based on the previously obtained results, an experiment was conducted in which 4 standards (S1 = 200 pg/μL, S2 = 100 pg/μL, S3 = 56 pg/μL, S4 = 28 pg/μL) were applied to the plate, along with sera diluted 2-fold and 4-fold. Based on the results obtained, two standard curves for human native neutrophil elastase were created, with values obtained in reaction with primary antibodies diluted 220-fold or 440-fold. The progress of both curves is shown in Figure 11.

**FIGURE 12:**
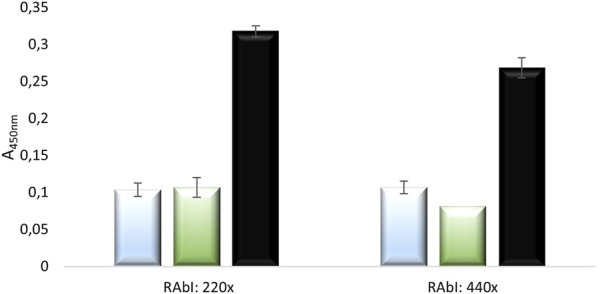
The values (corrected for control values) of absorbance for selected blood serum samples diluted 2-fold, 4-fold, and BLANK treated with primary antibody solutions diluted 220 times (RAbI:220x) or 440 times (RAbI:440x). The absorbance values for the 2-fold diluted serum are marked in light blue, and for the 4-fold diluted serum in green.

Based on the obtained results, it can be concluded that the construction of a standard curve illustrating the increasing relationship between absorbance values and NE standard concentrations (corrected for corresponding control absorbance values) was possible for standard samples treated with a 440-fold dilution of primary antibodies. The resulting standard curve for standards corrected with their corresponding control values for concentrations ranging from 28 pg/μL to 200 pg/μL, treated with 440-fold diluted primary antibody solution, shown in Figure 11 in orange, demonstrated a very high R^2^ value (0.987), indicating that the curve is well fitted to the progression of the function. It was also observed that the curve progresses within the range of mean corrected absorbance values from 0.312 to 0.505, which is within the desired absorbance range. The average absorbance value for the BLANK was 0.318 for the 220-fold dilution of primary antibodies and 0.27 for the 440-fold dilution of primary antibodies, as shown in Figure 12. Furthermore, Figure 12 presents the absorbance values (corrected for control values) of blood serum diluted 2-fold (blue) and 4-fold (green) treated with primary antibodies diluted 220 times (RAbI:220x) or 440 times (RAbI:440x).

Based on the obtained results, it was concluded that all absorbance values of the serum samples, corrected for the corresponding control absorbance values, were lower than the absorbance values of the BLANK. The calculated standard deviations indicated a high reproducibility of the results. However, the patient serum sample contained a relatively low concentration of NE. Given the relatively high BLANK values compared to all other control samples, i.e., serum samples treated only with secondary antibodies, for which the average absorbance value was << 0.1, and standard samples treated only with secondary antibodies, for which the average absorbance value was also << 0.1 (even for samples containing trace amounts of human native neutrophil elastase), it was inferred that the reaction conditions for the BLANK were not comparable to those for the other control reactions. The BLANK likely favored the reaction of secondary antibodies with the well walls coated with primary antibodies—unlike other wells, which were not treated with any analyte containing even trace amounts of protein. Additionally, the incubation time with the blocking factor was short, which may have resulted in incomplete protein coverage on the well, allowing primary antibodies to bind to the well walls and consequently facilitating the reaction of secondary antibodies with the bound primary antibodies. Therefore, it was concluded that in the next experiment, the blocking of wells corresponding to the BLANK should occur during the antigen (standard or serum) application stage to extend the incubation time with the protein.

In the next experiment, a 96-well plate was coated with standards (S1- 200, S2- 150, S3- 100, S4- 75, S5-50, S6- 25, S7- 14 [pg/μL]), 14 randomly selected samples from 200 blood serum samples, diluted 2-fold, and primary antibodies at a dilution of 1:440. Control wells for secondary antibodies were included for both the standards and the serum samples. To determine the detection range of the reference standard, recombinant neutrophil elastase was diluted in PBS in the following order: S1 - 200, S2 - 150, S3 - 100, S4 - 75, S5 - 50, S6 - 25, S7 - 14 [pg/μL], following a 1:2 dilution series. This allowed for the generation of a seven-point calibration curve. The linearity of the assay was determined based on the coefficient of determination (R2) for the entire concentration range and residual analysis (examining the residuals of the regression). Deviations from linearity (percentage deviation of measured values from the regression line) were also determined and did not exceed the ±15% threshold. The lower limit of quantification (LLOQ) was defined as the lowest calibrator concentration, while the upper limit of quantification (ULOQ) was defined as the highest calibrator concentration. Both parameters could be reliably determined with a coefficient of variation (CV) < 20%. The seven-point calibration curves covered the assay’s dynamic range and could be used to quantify samples with unknown NE concentrations. Intra-assay precision was calculated according to three samples with low, middle and high level of NE tested 15 times on one plate, respectively was calculated according to three samples with low, middle and high level of NE tested on three different plates, seven replicates in each plate. The following formula was used for the calculation:

$$CV\;\left(\%\right)=\frac{SD}{\text{mean}}\times 100$$

The intra-assay precision was 7%, while the inter-assay precision was 17%. Inter-day precision and accuracy were assessed over three randomly selected days. It was assumed that the relative standard deviation (RSD) of back-calculated values for each calibrator must be ≤ 15%, and the relative error (%RE) of back-calculated values for each calibrator must fall within the range of ±15% RE, as shown in Table 1.

**TABLE 1:** Accuracy results for each calibration point (200–14 pg/μL).

Nominal concentration (pg/µL)	Measured concentration (pg/µL)	RE [%]	Recovery [%]
200	198.2	−0.9	99.1
150	147.1	−1.93	98.07
100	101.3	1.1	101.1
75	71.2	−5.06	94.93
50	47.8	−4.4	95.6
28	31.2	11.43	111.43
14	15.8	12.9	112.9

**FIGURE 13:**
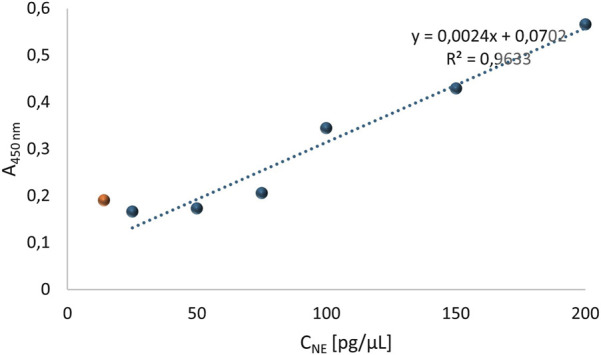
Standard curve for human native neutrophil elastase determined based on absorbance values obtained using primary antibodies diluted 440 times, in the concentration range of standard: 14 pg/μL to 200 pg/μL. The main steps of the ELISA test were carried out under the following conditions: I) Incubation with AbI: 440x, 2.5 h, 37°C; II) blocking solutions: 7.5% skimmed milk, overnight, 4°C; III) AbII: 5000x, 1.5 h, 37°C.

Based on the obtained results, a standard curve for human native neutrophil elastase (NE) was created. The progress of the curve is shown in Figure 13. The standard curve for the standards corrected by their corresponding control values in the concentration range of 14 pg/μL to 200 pg/μL, using primary antibodies diluted 440 times, exhibited a very high determination value (R^2^ = 0.963), indicating that the curve is correctly fitted to the progression of the function. Furthermore, it was noted that the curve progressed within the range of mean corrected absorbance values from 0.11 to 0.505, which is the desired range of absorbance values. The readout for NE concentration in the patient serum samples was possible for absorbance values starting from 0.11, although the intersection point of the line was at 0.0702 – below this value, disturbances in the results were observed, as seen for the standard with a concentration of 14 pg/μL (point marked in orange in Figure 13).

**FIGURE 14:**
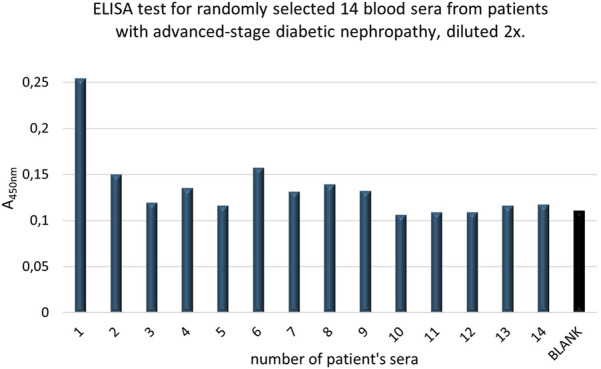
The mean absorbance values of fourteen randomly selected serum samples from patients with advanced diabetic nephropathy (stage IV or V), who had recovered from COVID-19, and the background (BLANK) obtained in the designed ELISA test for quantitative assessment of NE in blood serum.

Before determining the NE concentrations in the serum samples, it was checked whether the obtained mean absorbance values were lower than the average absorbance value for the BLANK. The results are shown in Figure 14. Based on the results presented in Figure 14, it was determined that the mean absorbance values for all 14 randomly selected serum samples from patients with advanced diabetic nephropathy were higher than the mean value of the BLANK, which was a key factor in optimizing the conditions for the designed ELISA test. Next, using the standard curve shown in Figure 14 and the average absorbance values (4 replications) of the serum samples (A) corrected by the average absorbance values of the control samples (A_K_), the NE concentrations in the patients’ serum samples were determined. To confirm the reliability of each result, the signal-to-noise (S/N) ratio was calculated, where noise was the random fluctuation of the signal, and the signal represented the net response for the sample. Results were considered reliable when S/N > 2.0. The results are presented in Table 2.

**TABLE 2:** Determination of NE concentration in the 14 randomly selected serum samples from patients with advanced nephropathy who have recovered from COVID-19.

	A	A_K_	A-A_K_	C [pg/µL]	C x R [pg/µL]	C x R [pg/mL]	S/N
1	0.254	0.082	0.172	42.42	84.83	84833.33	3.10
2	0.15	0.060	0.09	8.25	16.50	16500.00	2.50
3	0.119	0.066	0.053	0.00	0.00	0.00	1.80
4	0.135	0.064	0.071	0.33	0.67	666.67	2.11
5	0.116	0.066	0.05	0.00	0.00	0.00	1.76
6	0.157	0.063	0.094	9.92	19.83	19833.33	2.49
7	0.131	0.062	0.069	0.00	0.00	0.00	2.11
8	0.139	0.070	0.069	0.00	0.00	0.00	1.98
9	0.132	0.057	0.075	2.00	4.00	4000.00	2.32
10	0.106	0.075	0.031	0.00	0.00	0.00	1.41
11	0.109	0.078	0.031	0.00	0.00	0.00	1.40
12	0.109	0.104	0.005	0.00	0.00	0.00	1.04
13	0.116	0.117	0	0.00	0.00	0.00	0.99
14	0.117	0.166	0	0.00	0.00	0.00	1.41

Based on the conducted ELISA test, it was determined that the NE concentration could be measured in 5 out of 14 serum samples from patients (35.7%). In the remaining samples, the concentration was below the detection limit. It should be noted that physiologically, NE is practically absent in serum (or present at very low concentrations), and it is found inside neutrophils, appearing only upon their damage (as an enzymatic marker). The developed conditions of the ELISA test allow for effective determination of NE concentration in serum samples.

Next, the test was applied to 200 serum samples from patients, who were divided into the following groups: (1) Patients with T2DM from whom sera were collected before COVID-19, i.e., in 2019/2020 (n = 40 samples); (2) Patients with T2DM from whom sera were collected after recovering from COVID-19 (n = 40 samples); (3) Patients with an advanced stage of nephropathy (most commonly diabetic nephropathy), i.e., stage IV or V, from whom sera were collected before COVID-19 (n = 40 samples); (4) Patients with an advanced stage of nephropathy (most commonly diabetic nephropathy), i.e., stage IV or V, from whom sera were collected after recovering from COVID-19 (n = 40 samples); (5) The control group (n = 40 samples). The obtained values were comparable to those obtained using the commercial test. A comparative analysis will be presented in future publications.

To determine the selectivity of the ELISA test, a control sample containing only recombinant human neutrophil elastase (standard) and a sample containing recombinant human neutrophil elastase along with human albumin (a potential interfering factor) at a concentration of 5.5 g/dL were analyzed. Selectivity was calculated as the ratio of NE concentration in the interfering sample to the NE concentration in the control sample. The test was performed in five replicates. The average of the obtained values was 1.002 (100.2%), with a standard deviation of 0.003. It was concluded that the test demonstrates a high level of selectivity.

Additionally, the Limits of Detection (LoD) were determined, calculated according to formula:

$$LoD=\frac{Sm-Sblank}{slope}$$

Sm – minimum analyte signal

Sblank – mean blank signal

slope – the minimum/maximum concentration or mass of analyte detectable at a specified confidence level

LoD – limits of detection.

The LoD value was at least 36.23 pg/mL and was considered the detectable concentration in the designed ELISA test.

## 4 Discussion

In the last half-century, various ELISA methods have been developed to determine selected analytes in different types of biological materials such as blood serum, plasma, urine, stool, and cerebrospinal fluid. Although there are various configurations of these tests, such as the “sandwich” type, “double sandwich” type, and competitive tests, they are generally divided into two main types: discrete reading and continuous reading (which require calibrated standard curves). Discrete reading allows for simple quantitative assessment, although comparing results obtained in different laboratories can be problematic. Continuous reading tests are more commonly used because the calibrated standard curve prepared for each plate enables comparison of results across different laboratories. These results can be expressed as the concentration of the analyte under investigation (µg/mL), arbitrary ELISA units (EU/mL), or international units (IU/mL) for tests calibrated against international standards. However, a disadvantage of these tests is the need for a so-called representative standard serum.

This study also aimed to develop a solid-phase immunoenzymatic test (ELISA) for the quantitative assessment of NE in human blood serum. It was therefore necessary to select analytes in such a way that the test would be specific, and the control of each stage would confirm its reliability. The standard selected was native human neutrophil elastase obtained from human neutrophils, which was tested at concentrations ranging from 1 pg/μL to 300 ng/μL against various dilutions of primary antibodies (anti-human NE) such as 220x, 440x, 880x, and 2200x, as well as different blocking solution configurations commonly used in ELISA tests, such as skimmed milk, bovine serum albumin solution, and casein. Similar tests were performed on various concentrations (2x, 4x, 8x, 10x, 25x, and 50x) of several serum samples from patients with advanced diabetic nephropathy who had recovered from COVID-19. It was determined that the standard curve would be six-point in the concentration range of 25 pg/μL to 200 pg/μL, with patient blood serum being diluted 2 times. Both the standard and patient serum samples were each applied to the plate in duplicate, which is a universally accepted procedure. The curve shape was adjusted based on the R^2^ value (Aruta et al., 2023). The developed test conditions allowed for the creation of a line with an R^2^ value close to one (i.e., 0.963). The BLANK was considered to be the absorbance of wells treated with 7.5% skimmed milk, which were applied in parallel with the wells containing antigen solutions (i.e., standard or patient serum solutions).

An important step influencing the absorbance values was the blocking stage of the plate before applying the primary and secondary antibody solutions. Various concentrations of three blocking agents were tested: skimmed milk (5%, 7.5%, 10%, 12.5%), bovine serum albumin solution (0.5%, 1%, 1.5%, and 2%), and casein (0.5%, 1%, 1.5%). It was shown that only skimmed milk solutions did not cross-react with primary antibodies. Absorbance values higher than 0.1 were observed for the other blocking agents, indicating non-specific reactions with primary antibodies. Based on the tests, it was concluded that the most optimal blocking condition was a 7.5% skimmed milk solution.

To optimize the primary antibody application step, different dilutions of these antibodies were analyzed: 220x, 440x, 880x, and 2200x, with standard samples at different dilutions and patient serum samples from advanced nephropathy patients at various dilutions. The primary antibodies used were purified antibodies specific for human neutrophil elastase, produced as mouse, unconjugated IgG1, which had been successfully used by other research team (Floyd et al., 2016). A control for this stage was conducted by performing the ELISA test on parallel samples without primary antibody treatment. This aimed to confirm that no color change occurred in the sample (to exclude the possibility of absorbance due to interactions between primary antibodies and the secondary antibody substrate through antigen reaction). The conducted tests confirmed the absence of cross-reactivity of primary antibodies with the secondary antibody substrate.

The secondary antibody application step was also controlled, which involved applying the wells with the given antigen at the specified concentration only with secondary antibodies. It was noted that for each type of antigen (i.e., standard solution or patient serum), the absorbance values of these control samples were <0.07, confirming the absence of cross-reactions of secondary antibodies with antigens. The secondary antibodies were chosen to be specific for mouse IgG1, with the manufacturer guaranteeing no cross-reactivity with human IgG1. These antibodies had been successfully used by other research teams (Cui et al., 2024; Vasileva et al., 2024). The antibodies were dissolved in PBS, which is also a common practice (Bronowicka-Szydełko et al., 2021; Aruta et al., 2023).

The preliminary designed ELISA test for NE determination exhibited a relatively high sensitivity, initially set at 40 pg/μL (considering the dilution of the patient serum), and a reading range of 50 pg/μL to 400 pg/μL (CV < 30%), though this requires confirmation through testing on multiple plates. The absorbance values in patient serum samples fell within the test range, but the test would need to be applied to a significantly larger number of patient serum samples from individuals with various metabolic diseases. However, based on the preliminary results, it can be concluded that the test conditions and selection of analytes are correct, and the controls for each test stage are valid, with reproducible results.

The determination of NE concentration using ELISA has so far been conducted by research teams from various regions of the world, using different types of biological materials in various diseases (Allam et al., 2018; Narasaraju et al., 2024). Studies conducted on a Vietnamese population showed that elevated NE concentrations occurred in the lungs of patients affected by bronchiectasis. The material evaluated was sputum, a sample strongly correlated with bronchoalveolar lavage fluid (BALF). NE concentration was determined using an ELISA kit (Human PMN (Neutrophil) Elastase ELISA Kit, ThermoFisher Scientific, Massachusetts, GA, USA). The detection range of this test is 0.16–10.0 ng/mL, sensitivity: 1.98 pg/mL, intra-assay CV: 4.8%, inter-assay CV: 5.6%. Studies conducted using this test on ascitic fluid (AF) from 45 Egyptian patients with chronic liver diseases showed an accuracy of 70% (Nguyen-Ho et al., 2025). Additionally, the determination of NE using this test in 73 pregnant women from Finland revealed that elevated NE levels were associated with intra-amniotic infection (IAI) and microbial invasion of the amniotic cavity (MIAC), with 100% sensitivity at thresholds established from ROC curve analysis (Myntti et al., 2017). A blinded cohort study conducted by a French-Italian-Spanish team on a group of 120 patients with stage III colon cancer showed that NE levels were strongly associated with circulating DNA (a frequent marker used to guide targeted cancer therapies) (Mirandola et al., 2024). NE was determined using an ELISA kit (Human Neutrophil Elastase/ELA2 DuoSet ELISA, R&D System, DY008, DY3174, and DY9167-05). This test has a detection range of 46.9–3,000 pg/mL, though the datasheet does not provide information on its sensitivity, inter-assay, or intra-assay CV. NE levels measured in other studies using the same test were also found to be potentially significant in predicting cerebral vasospasm in patients with aneurysmal subarachnoid hemorrhage (aSAH) (Sajjad et al., 2024). These exploratory studies, conducted on a group of 62 patients with aSAH, 17 patients with unruptured cerebral aneurysms, and 12 healthy controls, showed that NE levels were significantly higher in patients with aSAH (84.8 ± 221.0 vs. 199.2 ± 218.9 ng/mL, p < 0.05) on the first day after aSAH, suggesting the involvement of NETs components in the pathophysiology of aSAH and subsequent events. Individually, NE levels on the first day after aSAH did not differ between patients with and without cerebral vasospasm. However, when combined in a logistic model, they enabled the prediction of cerebral vasospasm with high sensitivity (91%) and specificity (79%). The most recent study, conducted on a group of 319 individuals aged 45–89 years, with a 1:1 gender ratio, divided into five groups: (1) patients with T2DM whose sera were collected before COVID-19 (n = 59); (2) patients with T2DM whose sera were collected after recovering from COVID-19 (n = 62); (3) patients with T2DM and advanced nephropathy whose sera were collected before COVID-19 (n = 51); (4) patients with T2DM and advanced nephropathy whose sera were collected after recovering from COVID-19 (n = 55); and (5) a control group of voluntary blood donors whose sera were collected before the COVID-19 pandemic (average age: 51 years, n = 98), demonstrated that a significantly higher number of individuals with advanced nephropathy who recovered from COVID-19 exhibited elevated NE levels above 385 pg/mL (Group 4) compared to the same group before COVID-19 (Group 3) (Rabczyński et al., 2024). Interestingly, no differences were observed in NE levels among patients with T2DM without diabetic nephropathy between the pre- and post-COVID-19 groups. This suggests that NE may be an important marker of PCS in patients with advanced, chronic diabetic nephropathy. The test (Human Neutrophil Elastase ELISA Kit, cat. no. E0778Hu, SUNLONG BIOTECH CO (Hangzhou, China)) had the following selected parameter values: sensitivity >31 pg/mL, with a range of 78–5,000 pg/mL. Comparable NE values were noted in randomly selected patients using a custom-developed ELISA test, which is the subject of this study. The use of the custom ELISA test allows for the same conclusions as those derived from commercially available ELISA tests. Interestingly, the custom-designed test has a lower minimum detection range compared to many other tests (from 50 pg/mL to 200 pg/mL). It is approximately 200 times cheaper to conduct than a commercial test. Furthermore, it allows for control and optimization of each stage of the ELISA procedure, depending on the type of material being studied and the available equipment.

Acute COVID-19 illness can cause immunological, hematological, vascular, and even multi-organ changes in some individuals. To date, it has been shown that patients with post-COVID syndrome (PCS) exhibit persistent immunological changes, including reduced T cell subpopulations (CD4, CD8) and alterations in memory B cells (Guerrera et al., 2025). Dysregulation of platelet function and thrombotic issues associated with PCS have also been observed (Luzak et al., 2024). A systematic review of long-term COVID-19 symptoms lasting more than 6 months after the illness demonstrated that women have an increased risk of post-COVID-19 symptoms (risk ratio RR: 1.24, adjusted odds ratio 3.08). Conditions such as COPD/lung disease, overweight or obesity, hypertension, cardiovascular diseases, and asthma were identified as potentially linked to an increased risk of post-COVID-19 symptoms (Hill et al., 2024). Due to this heterogeneity, diagnosing and assessing the actual significance of PCS is challenging. Identifying patients at higher risk is also problematic. Demographic factors are not insignificant, as they highlight the dominance of different PCS causes across various populations (Hill et al., 2024). There is a significant need to identify molecular markers that could help in determining the cause of PCS, establish reliable methods for their identification, monitor disease progression, and guide treatment options. The goal of many research teams is to develop prognostic models for PCS based on parameters characteristic of PCS (which are being sought among those from the acute phase of COVID-19). Standard laboratory diagnostic markers are insufficient, as changes in their levels may be associated with other disorders. Therefore, efforts must be made to develop widely accessible methods for identifying potential markers specific to COVID-19 and PCS. One such marker is neutrophil elastase (NE), an enzyme released during excessive neutrophil activation from neutrophil extracellular traps (NETs) in the acute course of viral diseases, including COVID-19 (Gunasekara et al., 2024). NE contributes to the release of potent inflammatory mediators leading to multi-organ damage, and NE itself, in reaction with the viral spike protein, may lead to amyloidosis, considered one of the complications associated with PCS (Nyström and Hammarström, 2022). It has also been observed that elevated NE levels were more frequently found in individuals with advanced-stage diabetic nephropathy (stage V) who had symptomatic COVID-19 compared to patients with stage V diabetic nephropathy sampled before the COVID-19 pandemic. These observations underscore the necessity of developing methods for detecting markers such as NE, which would be accessible for examining patients living in different parts of the world.

In the next stages of the study, it is necessary to conduct a thorough statistical analysis examining the relationships between potential PCS markers, such as NE, and other standard laboratory diagnostic markers, diagnoses, and other potential PCS markers. These studies will be conducted on a group of 319 patients (with T2DM, with and without advanced diabetic nephropathy, before and after COVID-19, and a control group) from whom blood serum and plasma samples were collected. Various markers (both laboratory and clinical parameters) of inflammation, early vascular changes, oxidative stress, and selected compounds belonging to advanced glycation end products will be measured in the biological material. Understanding the interrelationships will primarily enable the identification of directions for further, more precise research aimed at uncovering the molecular mechanisms leading to their release into the bloodstream. In the next phase of the study, blood serum samples collected from patients with other conditions (e.g., schizophrenia, post-stroke, colorectal cancer, Alzheimer’s disease) will also be tested. Statistical analyses will be conducted in these groups, taking into account the values of new potential markers such as NE, standard laboratory diagnostic markers, diagnoses, and therapies applied.

## 5 Conclusion

The study attempted to independently develop an ELISA test for the quantitative assessment of NE content in patient blood sera. The biological material used in the study came from patients with advanced stages of nephropathy who had recovered from COVID-19. To optimize the developed method, various concentrations of the analytes were analyzed, including the protein standard (native human neutrophil elastase), the blocking agent (skimmed milk), primary antibodies specific to human NE, secondary antibodies specific to the primary antibodies, and the manufacturer’s guarantee of no cross-reactivity with human immunoglobulins. Controls were applied at each stage of the test to check for non-specific reactions.

It was determined that the standard curve, corresponding to concentrations of 400, 300, 200, 150, 100, and 50 pg/μL, allows for the quantitative assessment of NE in patient blood sera (initially set sensitivity: ≥40 pg/μL; CV < 30%). The optimal serum dilution was found to be 2-fold, primary antibody dilution: 440-fold, and secondary antibody dilution: 5000-fold. The most suitable blocking agent was found to be a 7.5% skimmed milk solution. Pilot studies conducted using the developed test showed that it was possible to detect NE in five out of 14 blood sera samples, and the NE concentrations in the biological material were within the test range.

The obtained values of the parameters characterizing the designed ELISA test (specificity, accuracy, the intra-assay and inter-assay precisions, LoD, LLOQ, ULOQ) indicate that this test is of a potential diagnostic value. The values of the obtained NE concentrations for the patient sera samples were comparable with the NE concentration values determined for the same sera samples using the ready-made ELISA test described in (Rabczyński et al., 2024). The cost of performing the designed test is one of the main merits for using that test. The said cost is significantly (approx. 200-fold) lower than the cost of performing a ready-made ELISA test. Moreover, the advantage of performing a self-designed ELISA test is that it is possible to control each stage of the test, as opposed to the performing a ready-made ELISA test in case of which the control over the whole process is hindered. Both standard curves in both tests are comparable. However, due to the fact that the test performance is characterized not only by the values of specificity, accuracy, or sensitivity of the test, but also by the positive and negative predictive value of the test (true relationships of patients to patients indicated in the test and falsely sick to healthy indicated in the test) in a given disease, it is not currently possible to determine the performance of ELISA tests detecting elevated NE concentration in PCS. This is due to the fact that PCS is a relatively new, little-known diagnosis, and it is practically impossible to reliably indicate which patient actually has PCS and in whom the disease begins initiated by a factor other than COVID-19. The actual key markers of early damage caused by COVID-19 indicating the development of PCS are still not known. NE is considered such a potential diagnostic marker important in PCS, and the designed ELISA test - hence the relevance of the assay test featured in this manuscript in context of PCS. Further studies are planned to conduct statistical analyses along with multivariate analyses, allowing for the verification of the relationships between NE concentrations and routine laboratory and imaging diagnostic parameters and diagnoses, with an additional context of the therapies used. However, cautious conclusions should be drawn, as the test still requires verification on a much larger group of subjects.

## Data Availability

The raw data supporting the conclusions of this article will be made available by the authors, without undue reservation.
